# Central Hemangioma of Mandible: Rare Case

**DOI:** 10.1002/ccr3.70892

**Published:** 2025-10-12

**Authors:** Rakshya Shrestha, Reena Kumari Shrestha, Dipti Shrestha, Alok Sagtani, Pranay Shakya

**Affiliations:** ^1^ Department of Oral and Maxillofacial Surgery People's Dental College and Hospital Kathmandu Nepal; ^2^ Department of Oral and Maxillofacial Surgery Kathmandu Medical College Kathmandu Nepal

**Keywords:** benign tumor, central hemangioma, intraosseous hemangioma, mandible, vascular anomalies

## Abstract

Central hemangioma, though it is a rare entity, should always be considered in the differential diagnosis of intraosseous radiolucent lesions, especially in younger patients, as missed diagnosis can lead to profuse intraoperative bleeding and pose life‐threatening complications. Comprehensive radiographic assessment and confirmatory biopsy are crucial for definitive diagnosis, surgical planning, and effective management.

## 1 Introduction

Hemangioma is a true benign neoplasm of endothelial origin. It is frequently encountered in soft tissues, presenting with swift postnatal expansion and slow spontaneous regression [1, 2]. Central hemangioma, also known as intraosseous hemangioma, occurs more frequently in the vertebral column and skull bone [3, 4, 5]. It is relatively uncommon in the mandible and maxilla, comprising only 0.5%–1% of all intraosseous tumors [4, 5, 6].

Although the mucosal and soft tissue lesions can easily be suspected based on their clinical presentation, it may be quite challenging to visually discern the intraosseous lesions. Hence, hemangioma poses both a diagnostic and therapeutic challenge [5, 6]. If not diagnosed and managed on time, hemangioma might cause massive bleeding, which can be a life‐threatening situation [7].

## 2 Case History/Examination

A 12‐year‐old female presented to the department of oral and maxillofacial surgery with a chief complaint of swelling in the lower right back region of the jaw for 5 years. The swelling was insidious in onset, progressive in nature, and was also associated with occasional bleeding gums. The patient reported the first episode of profuse bleeding 5 years ago following the extraction of a deciduous tooth, due to which the patient had undergone a blood transfusion. The patient also gave a history of pus drainage from the right side of the lower jaw 4 months ago, which subsided after a course of oral antibiotics. The patient reported being diagnosed as anemic 5 years ago. There was no family history of bleeding disorders.

**FIGURE 1:**
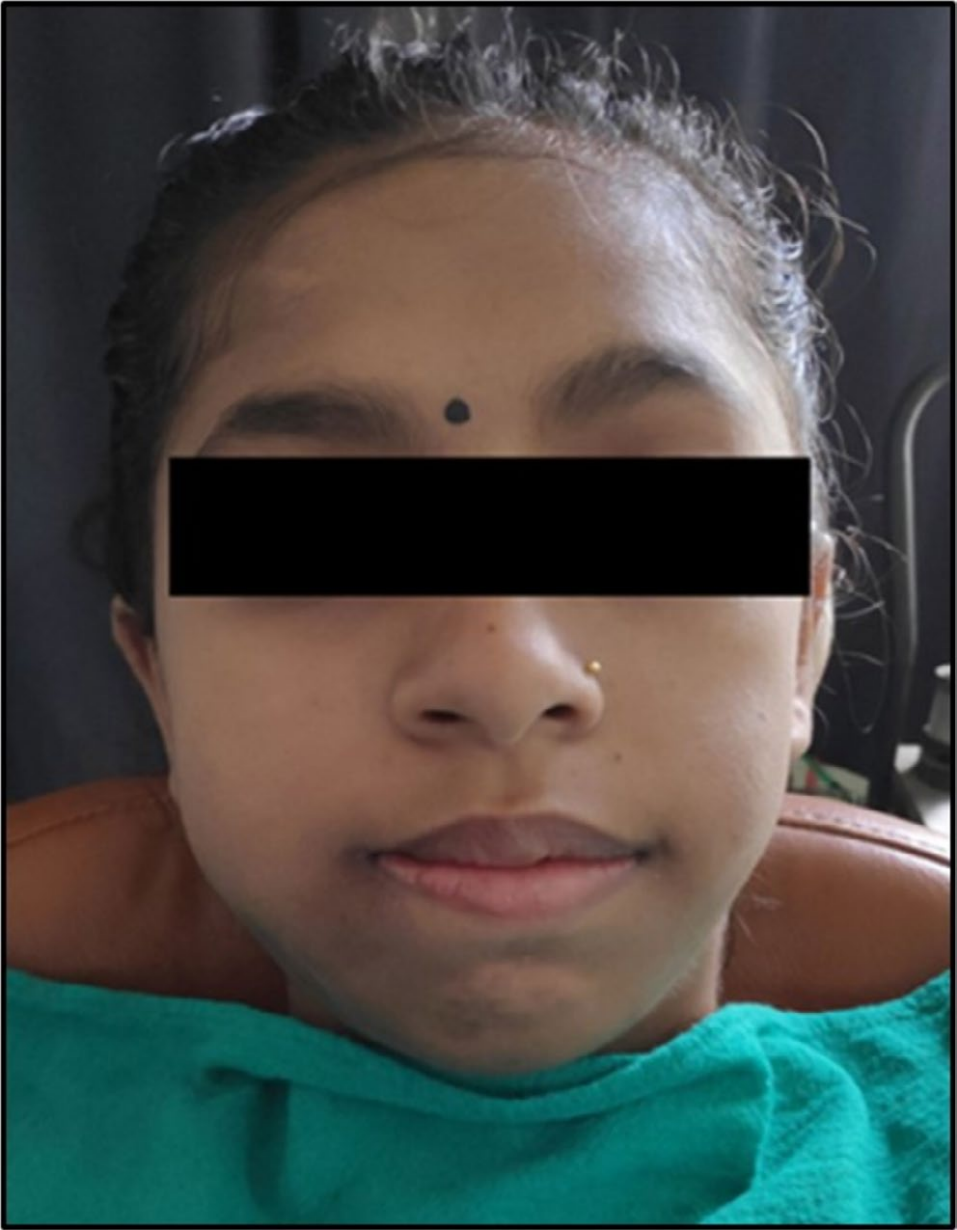
Extra‐oral examination revealing ill‐defined diffuse swelling extending antero‐posteriorly from right mandibular parasymphysis to angle of mandible and superoinferiorly from zygomatic arch to lower border of mandible.

Extra‐oral examination revealed an ill‐defined diffuse swelling on the right side of the mandible of size approximately 5 × 4 cm^2^, extending antero‐posteriorly from the mandibular parasymphysis to the angle of the mandible, and supero‐inferiorly from the zygomatic arch to the lower border of the mandible (Figure 1). On palpation, the swelling was hard in consistency, non‐tender, and non‐fluctuant. An extraoral sinus opening with crusting of overlying skin was present in the body of the mandible on the right side.

On intra‐oral examination, an ill‐defined diffuse swelling was present in the vestibular area extending from the canine to the second molar on the right side of the mandible. An intraoral sinus opening was present on the buccal cortex of 46. On palpation, the swelling was hard in consistency, non‐tender, non‐mobile, and non‐fluctuant. The teeth were intact in the arch with no sign of depression in the socket or protrusion from the socket. There was no sign of paresthesia.

## 3 Methods (Differential Diagnosis, Investigations, and Treatment)

Based on history and clinical examination, a provisional diagnosis of central hemangioma of mandible was made. Differential diagnosis included arteriovenous malformation, ameloblastoma, odontogenic myxoma, and central giant cell granuloma.

**FIGURE 2:**
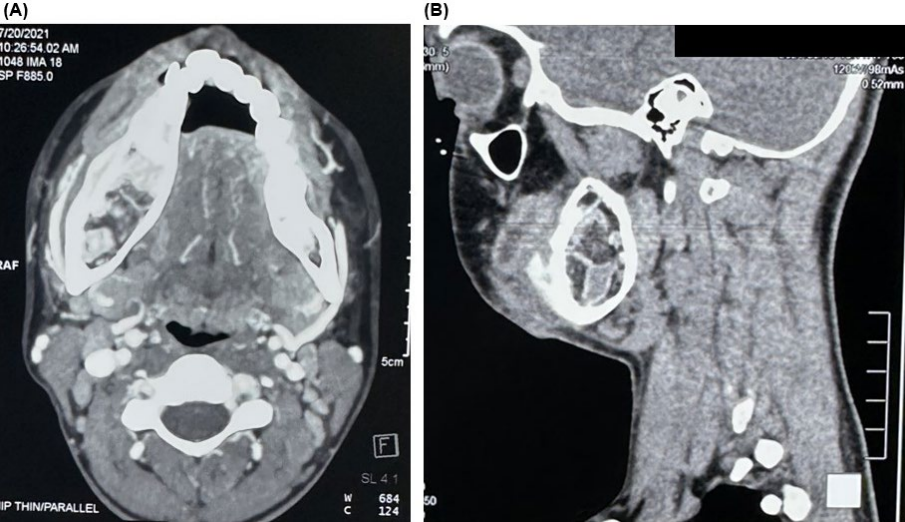
Computed tomography angiogram of mandible shows lytic expansion of body of right mandible and dilated right mandibular canal. (A): Axial view, (B): Sagittal view.

Computed tomography (CT) angiogram of the mandible revealed lytic expansion of the body of the right mandible with mild thinning and some areas of breach of the buccal cortex. Lamellated periosteal reaction adjacent to the buccal cortex was observed (Figure 2). Branches of the right inferior alveolar artery and right mandibular canal were dilated. An incisional biopsy was done, which was suggestive of central hemangioma. Correlating the CT angiogram reports with the incisional biopsy, a diagnosis of central hemangioma of the right side of the mandible was made, for which surgical resection was planned.

**FIGURE 3:**
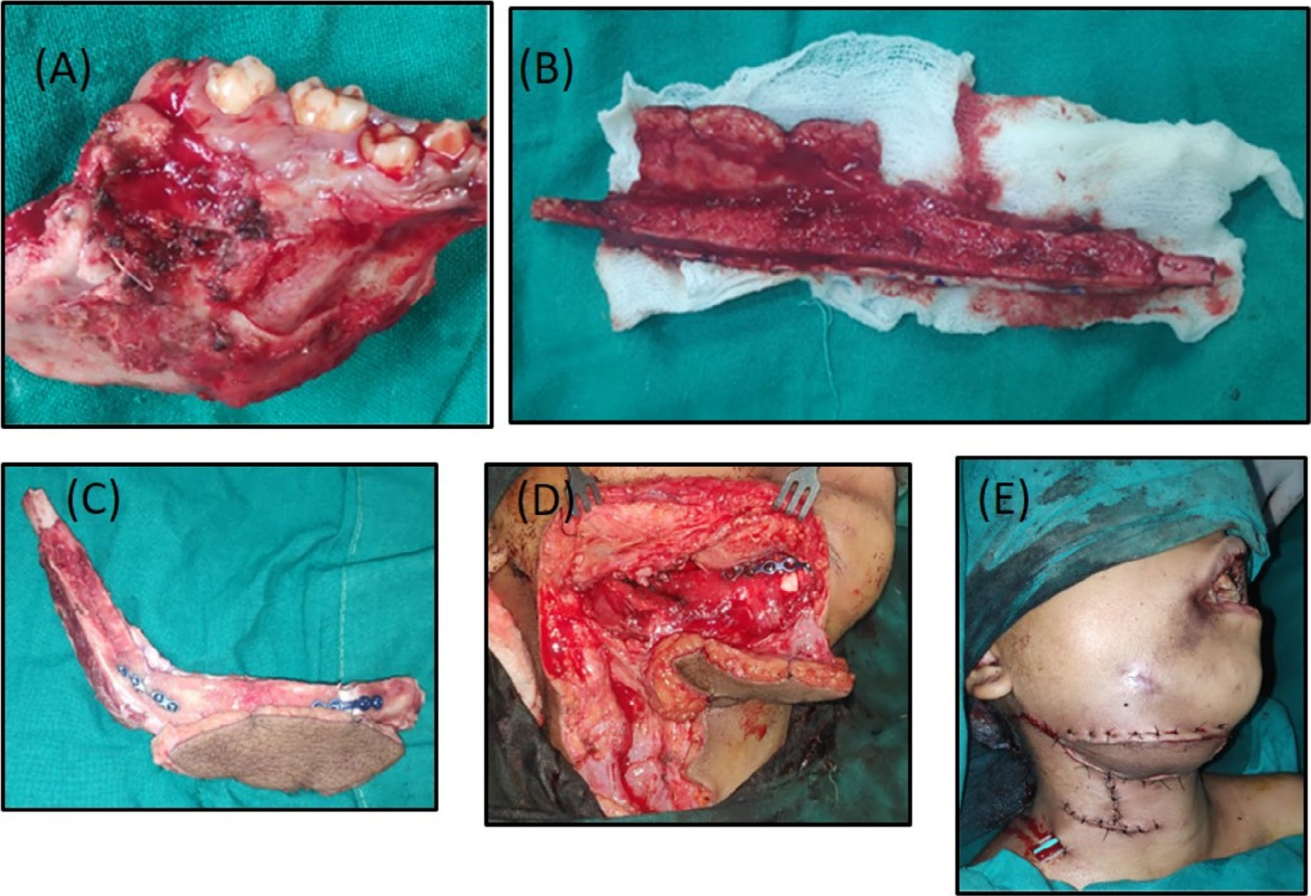
(A): Resected segment of mandible, (B): Free fibular flap harvested. (C): Single barrel free fibular flap reconstructed to the shape of resected mandible. (D): Stabilization of fibula flap with contralateral mandible using reconstruction plates and miniplates. Vascular anastomosis done. (E): Closure done in multilayer. Drain secured in place.

After discussion about the treatment plan with the patient party, written informed consent was obtained from the parents and assent was obtained from the patient. Segmental resection of the right mandible followed by primary reconstruction with a free fibular flap was carried out under general anesthesia (Figure 3).

## 4 Outcome and Follow‐Up

**FIGURE 4:**
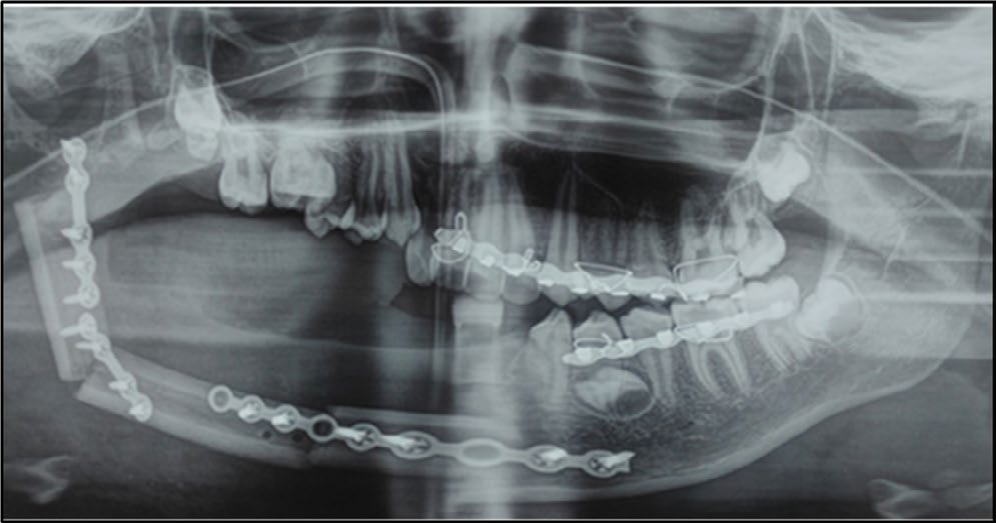
Immediate postoperative orthopantogram demonstrating adequate stabilization of free fibular flap using titanium plates and screws.

**FIGURE 5:**
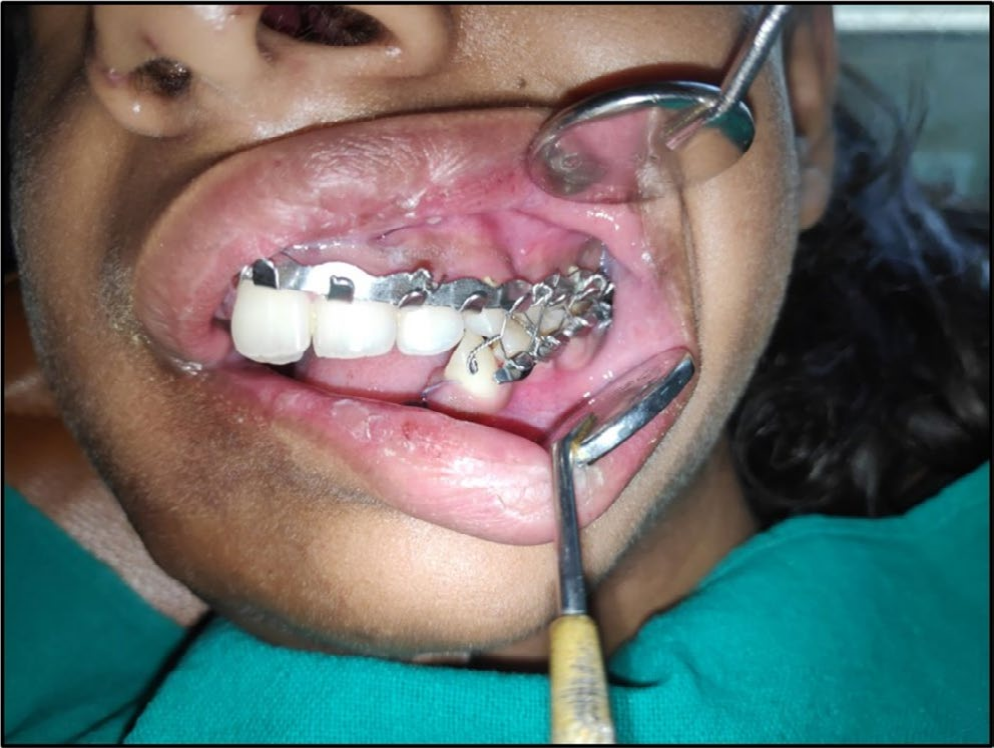
Intermaxillary fixation done postoperatively for 3 weeks for stabilization of reconstructed segments.

**FIGURE 6:**
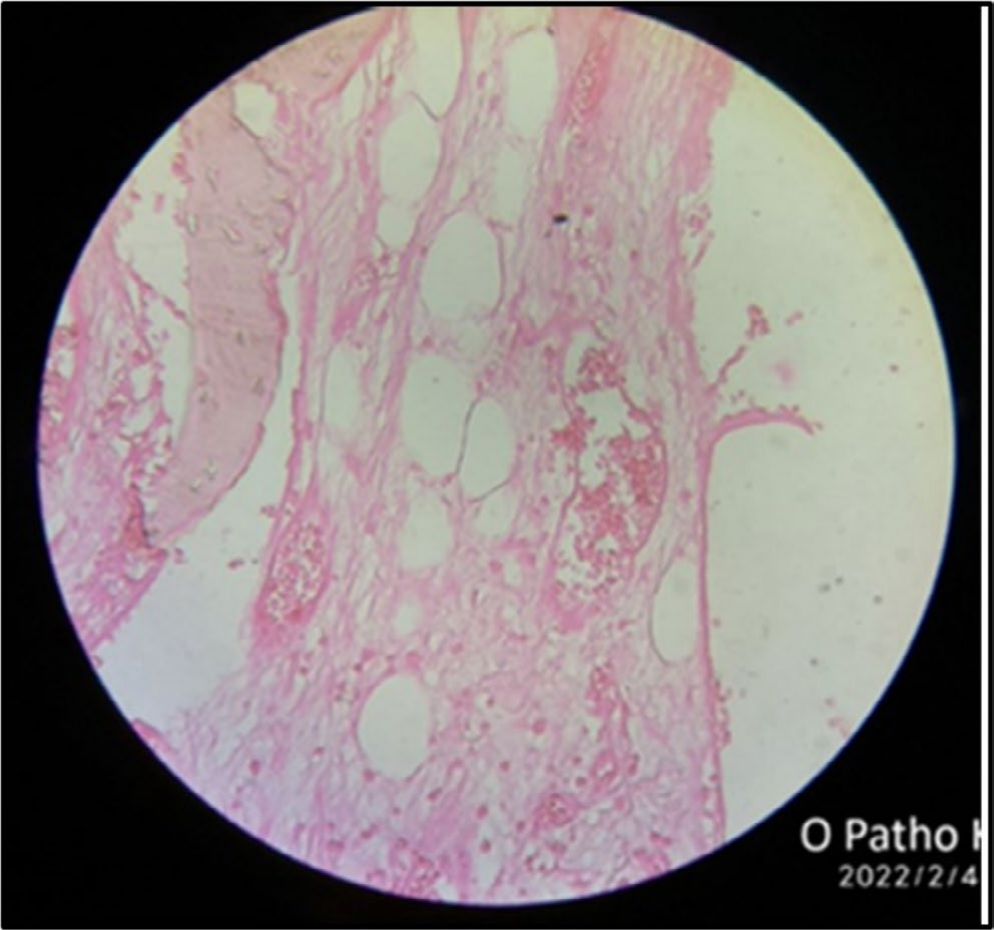
Histopathological report reveals the presence of multiple large sinusoidal spaces filled with RBC and surrounded by connective tissues. Areas of hemorrhage are also noted. Features suggestive of central hemangioma. (Hematoxylin and Eosin stained slide under 40× magnification).

**FIGURE 7:**
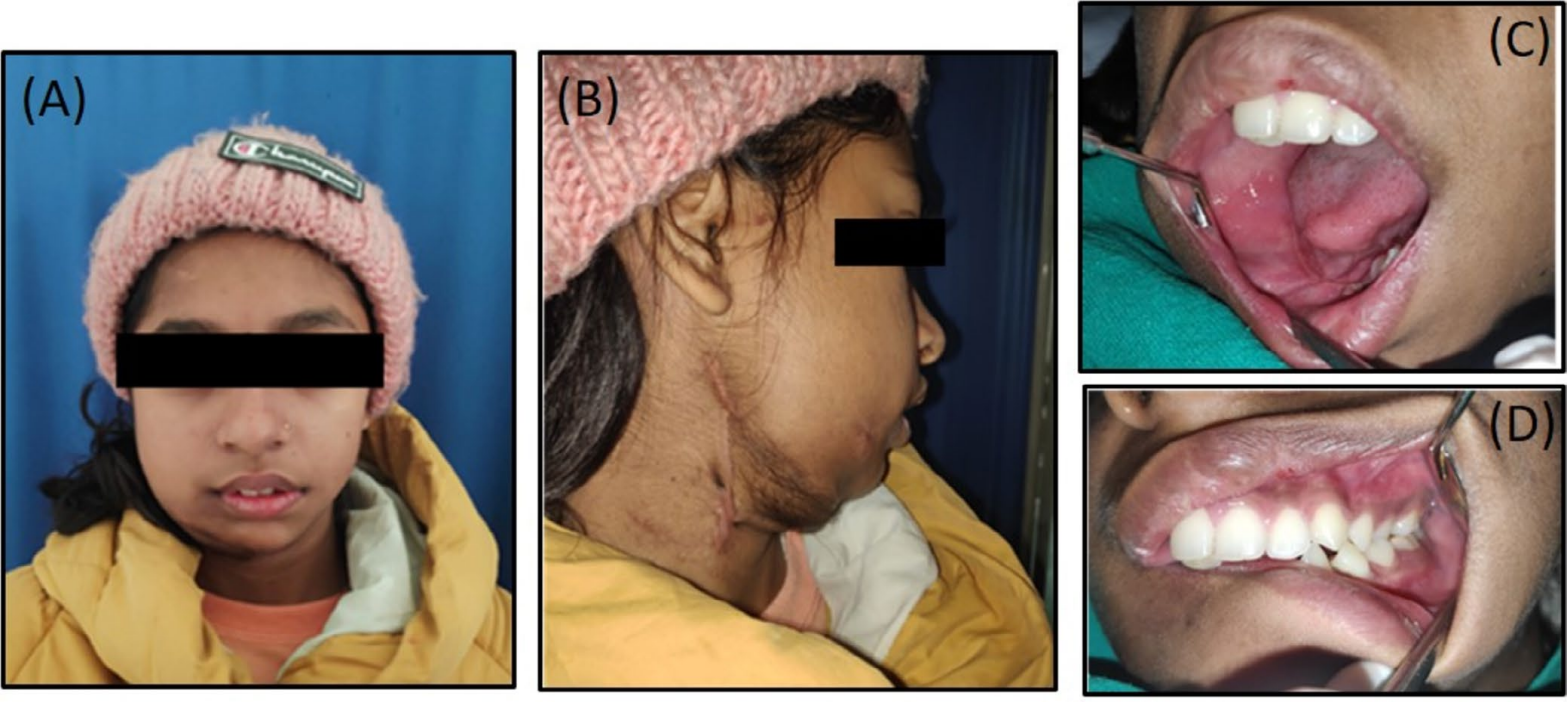
(A) Frontal view and (B) Right lateral view: Extra‐oral clinical picture revealed healed incision site at 1 year follow‐up. (C) and (D): Intra‐oral picture revealed healed intraoral resection and reconstruction site at 1 year follow‐up.

**FIGURE 8:**
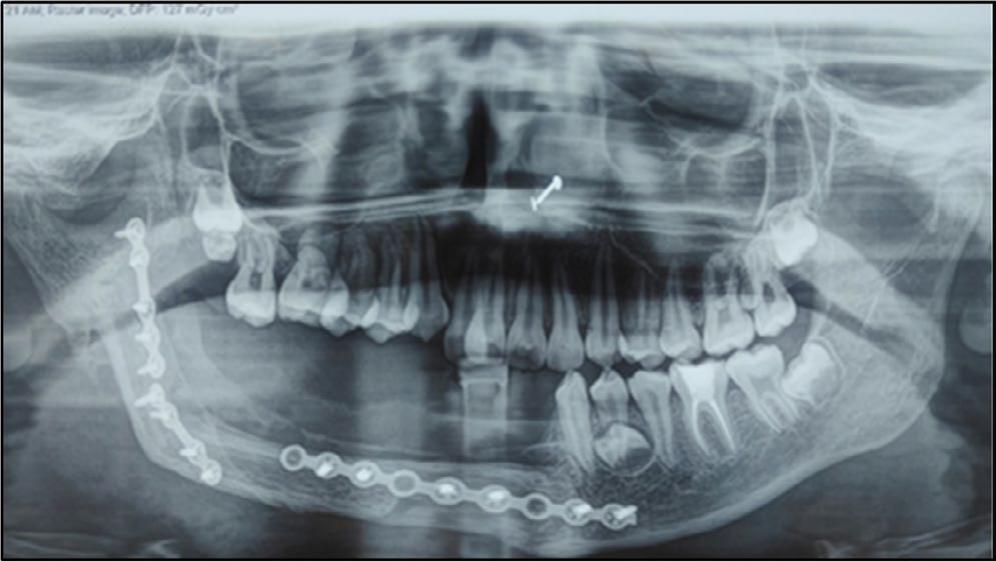
Orthopantogram revealed satisfactory osteosynthesis at 1‐year follow‐up.

Immediate postoperative orthopantogram was taken (Figure 4). Intermaxillary fixation (IMF) was done using arch bars for 3 weeks postoperatively in order to maintain the normal occlusion (Figure 5). The resected mandibular segment was sent for histopathological examination, which confirmed the diagnosis of central hemangioma (Figure 6). One year postoperative clinical picture and orthopantogram revealed satisfactory healing and osteosynthesis, respectively (Figures 7 and 8).

## 5 Discussion

The word hemangioma is derived from the Greek word, hema‐“blood,” angeio‐“vessel,” and oma‐“tumor.” The origin of central hemangioma is debatable [8]. It is considered a true benign neoplasm by some researchers because of the initial endothelial growth that eventually turns into blood vessels. Whereas, others claim it is a hamartoma brought on by the growth of intraosseous mesodermal cells that undergo endothelial differentiation and develop into canalized and vascularized tissue [9, 10].

Hemangioma is typically discovered during infancy. Most of the lesions regress or become involuted with increase in age up to puberty; whereas, sometimes the lesion increases in size and deteriorate resulting in life threatening situations. The true significance of the lesion lies in its potential to cause spontaneous bleeding which might at times be fatal [9].

Central hemangiomas are more prevalent in women than men (3:1), and the incidence peaks during the second decade of life [1]. The present case was reported in 12‐year‐old female child. Hemangiomas are more common in the mandible as compared to the maxilla. In the mandible, the lesion is more commonly seen in the body region, and rarely seen in the condylar region [1]. These results are consistent with the present case, which involved the posterior mandible.

Due to its intra‐osseous location, the lesion might be missed during the initial stage. The patient in this case also reported experiencing severe bleeding for the first time 5 years ago after extraction of a deciduous tooth, which led to the patient receiving blood transfusions. Although there are no distinctive clinical signs during the initial stage, some patients might present with painless, firm, bony enlargement that is occasionally accompanied by a pulsating or throbbing discomfort [6, 7, 10]. Other dental findings might include deranged occlusion, tooth displacement, supra‐eruption, premature primary tooth exfoliation, and early eruption of permanent teeth, which is caused by the pressure from a growing lesion [6, 8]. In the present case, painless firm swelling with sinus opening was the feature in consideration. Additionally, bruises or pulsation may be present, which signifies a rapidly growing lesion [8]. In some instances, epistaxis and spontaneous gingival bleeding from the affected area can also be seen. Incidences of uncontrollable bleeding have also been reported after biopsy or extraction [6].

Radiographs usually reveal patchy, unilocular or multilocular osteolytic lesions. Typically, three common patterns of central hemangioma have been described: sunray appearance, honeycomb or soap bubble appearance, and poorly defined radiolucency. There might be an apparent widening of the inferior alveolar canal. Other signs of a growing lesion include displacement of teeth or resorption of the roots of nearby teeth [11].

Radiographically, hemangioma resembles ameloblastoma, multiple myeloma, osteosarcoma, fibrous dysplasia, central giant cell granuloma, dentigerous cyst, or odontogenic cyst. Clinically, it resembles central arteriovenous fistula, aneurysms, or shunt [6]. Since central hemangioma is a great mimicker, definitive diagnosis and fabrication of a treatment plan rely heavily on the patient's history, radiography, and histopathology [6]. Definitive diagnosis of hemangioma is made by histopathological examination [6]. Angiography could also be a useful adjunct for the diagnosis, as it illustrates the pressure of the vascular lesion and helps to delineate the boundaries of lesions and arterial connections [6]. Recently, digital subtraction angiography (DSA) has proven to be a remarkable tool for the diagnosis of hemangioma, as DSA allows the visualization of main feeding vessels to the lesion [12].

The greatest complication encountered during the management of hemangioma is exsanguinating hemorrhage. Thus, the practitioner's main considerations when opting for a treatment strategy for central hemangioma should include control of hemorrhage, elimination of the lesion, and prevention of recurrence of the lesion. Various treatment modalities mentioned in the literature to date include noninvasive radiotherapy, pharmacotherapy (beta blocker like propranolol and steroids), injection of sclerosing agents, embolizing agents, and surgical intervention with curettage or radical resection [3, 11, 13].

Although most approaches have depicted moderate success statistically, surgical resection with safety margin either alone or in combination with embolization has been considered the most favored form of treatment despite the high risk of hemorrhage and functional impairment [3, 8, 10, 13]. In the present case, surgical resection with adequate margin followed by immediate reconstruction with free fibular flap was opted considering the tumor size, progression rate, child growth potential, and tumor approachability. Radiotherapy was avoided considering the complications of radiation in growing children. Also, sclerosing agent did not have much role due to the intraosseous nature of the lesion. However, the authors believe that if the feeding vessel had been embolized, intraoperative hemorrhage could have been significantly reduced. Virtual surgical planning and a double‐barrel flap with customized titanium plate and immediate implant insertion would have been preferable in the present case, but due to the financial constraints this treatment plan could not be implemented.

## Author Contributions

**Rakshya Shrestha:** writing – original draft, writing – review and editing. **Reena Kumari Shrestha:** writing – review and editing. **Dipti Shrestha:** writing – review and editing. **Alok Sagtani:** writing – review and editing. **Pranay Shakya:** supervision, writing – review and editing.

## Consent

Patient's written informed consent was obtained for publication of this case report and accompanying images. A copy of the written consent is available for review by the Editor‐in‐Chief of this journal on request.

## Conflicts of Interest

The authors declare no conflicts of interest.

## Data Availability

All relevant clinical, radiological, and histopathological data supporting the findings of this case report are included within the article.
